# Adsorption behavior of furan at Ge(100) surface

**DOI:** 10.1038/s41598-023-34843-z

**Published:** 2023-05-19

**Authors:** Jeong-Woo Nam, Han-Koo Lee, Byeong-Seon Kim, Jin Seog Gwag, Youngsoo Kim, Young-Sang Youn

**Affiliations:** 1 Department of Chemistry, Yeungnam University, Daehak-ro 280, Gyeongsan, Gyeongbuk 38541 Republic of Korea; 2 Pohang Accelerator Laboratory, Pohang University of Science and Technology (POSTECH), Pohang, 37673 Republic of Korea; 3 Department of Chemistry Education and RINS, Gyeongsang National University, Jinju, 52828 Republic of Korea; 4 Department of Physics, Yeungnam University, Daehak-ro 280, Gyeongsan, Gyeongbuk 38541 Republic of Korea

**Keywords:** Surface assembly, Surface spectroscopy

## Abstract

The adsorption behavior of furan on the Ge(100) surface was studied using a combination of high-resolution photoemission spectroscopy (HRPES) and density functional theory (DFT) calculations. We identified the two adsorption species produced by the [4 + 2] cycloaddition and deoxygenation reactions of furan with the Ge(100) surface in a ratio of approximately 76:24 at the surveyed coverages, via an analysis of the binding energies and relative area proportions of all the peaks in the C 1*s* and O 1*s* core-level spectra. The DFT simulation results revealed that the [4 + 2] cycloaddition and deoxygenation adducts are thermodynamically preferred by the reaction of furan with the Ge(100) surface compared with others, which is consistent with the HRPES results. The findings will further our understanding of the surface reactions of five-membered heterocyclic molecules.

## Introduction

Group 14 semiconductor surfaces can serve as a platform capable of forming organic–semiconductor hybrid structures by reacting with organic molecules and thus can be used in molecular memories, electronics, and sensors^1–7^. However, to successfully form an organic–semiconductor hybrid structure, an in-depth understanding on the interactions that occur between the organic molecules and semiconductor surface is essential for tuning the semiconductor surface properties^8^.

Since a dimer on the reconstructed (100) surface of a Group 14 semiconductor simultaneously exhibits π-bonding and zwitterion characteristics, it can react with organic molecules via cycloaddition and Lewis acid–base reactions^9–13^. Because a molecule containing two C=C bonds, such as butadiene, can act as a conjugated diene, a five-membered aromatic molecule containing a heteroatom and two C=C bonds in the ring system can also serve as a conjugated diene and initiate a [4 + 2] cycloaddition reaction with the surface dimer of Ge(100) or Si(100) while functioning as a dienophile^14^. Five-membered aromatic heterocyclic molecules such as furan, thiophene, and selenophene undergo a [4 + 2] cycloaddition reaction on the Si(100) surface^15–18^. In particular, in the cases of thiophene and selenophene, desulfurization and deseleniumization structures are produced by the subsequent reaction of the formed [4 + 2] cycloaddition structure, wherein a heteroatom is transferred to an adjacent surface dimer^17,18^.

Of late, there has been a resurgence in the research interest in the Ge(100) surface because it exhibits several attractive properties such as a narrow bandgap, high electron/hole mobility, and a lower dopant activation temperature than that of the Si(100) surface^19–22^. In other words, the Ge(100) surface can be used as a transistor material with properties better than those of Si(100) for improving device performance^23^. Recently, germanium with the attractive properties was exploited to develop a promising silicon–graphene–germanium transistor for ultra-high frequency operation^24^. However, to effectively use Ge(100) in electronic devices, a high-quality oxide layer must be formed, as in the case of Si(100)^19^. Therefore, the interactions between oxygen-containing molecules and the Ge(100) surface must be elucidated. Furan (C_4_H_4_O) is the simplest oxygen-containing aromatic molecule, which plays a significant role as a structural subunit in various natural products and drug molecules^25,26^. While the adsorption properties of furan on the Si(100) surface have been investigated^15,16^, those on the Ge(100) surface remain unknown. Although the adsorption behavior of furan on the Ge(100) surface can be predicted solely using density functional theory (DFT) calculations, the accuracy of the simulated results is reduced when long-range van der Waals interaction is important, as reported in the literature^27,28^. Therefore, the reaction behavior of furan, which contains an oxygen atom, on the Ge(100) surface should be analyzed including the experimental technique to obtain fundamental information regarding the surface chemistry of the Ge(100) semiconductor.

In this study, we elucidate the adsorption structures of furan on the Ge(100) surface using high-resolution photoemission spectroscopy (HRPES) and DFT calculations. Based on the HRPES results and DFT data, we found that one of the structures of furan adsorbed onto the Ge(100) surface forms via a [4 + 2] cycloaddition reaction, as in the case of the Si(100) surface^15,16^. In addition to this [4 + 2] cycloaddition structure, another adsorption adduct is formed, which was confirmed to be a deoxygenation structure; this structure has not been reported in the case of the adsorption of furan onto the Si(100) surface.

## Experimental and computational methods

The HRPES experiments were performed at the 10A2 HR-PES II beamline of the Pohang Accelerator Laboratory. The surface of the Ge(100) crystal (n-type, Sb doped), which had dimensions of 3 mm × 14 mm, was cleaned via several rounds of Ar^+^ ion sputtering followed by annealing. The cleanliness of the Ge(100) surface was confirmed based on its O 1*s* and C 1*s* spectra. Furan (C_4_H_4_O, ≥ 99% purity, Sigma-Aldrich) was purified via repeated freeze–pump–thaw cycles to eliminate all the impurities before it was brought into contact with the Ge(100) surface through a leak valve. The C 1*s* and O 1*s* core-level spectra were recorded using a SCIENTA 2002 electron analyzer (Scienta Omicron, Germany) at the photon energies of 362 and 653 eV, respectively, with a pass energy of 20 eV and an energy step of 0.02 eV to improve the surface sensitivity. The base pressure of the ultrahigh vacuum chamber was maintained at less than 1.3 × 10^–9^ Torr. All the HRPES spectra were analyzed using the standard nonlinear least-squares fitting method with Voigt functions^29^; the binding energies of the observed peaks were calibrated with respect to that of the Au 4*f*_*7/2*_ peak (84.0 eV), as measured using a Au foil at the same photon energy.

The geometrically optimized adsorption structures and corresponding energies of furan on a four-dimer (Ge_35_H_32_) cluster model passivated by H atoms were obtained via DFT calculations performed using Becke’s three-parameter non-local exchange functional with the Lee–Yang–Parr (B3LYP) correlation functional and a basis set at the LACVP** level^30^, in the JAGUAR 10.1 software package. LACVP** is a mixed basis set of LAV3P for Ge atoms and 6–31G for atoms from H to Ar, where the elements beyond Ar in the periodic table are described by the Los Alamos effective core potentials (ECPs) developed by Hay and Wadt^31^. For the four-dimer cluster used in the DFT calculations, the three bottom-most Ge layers were frozen at the experimentally determined reconstruction positions of the Ge(100)-c(4 × 2) surface to inhibit the unphysical Ge cluster geometries^32,33^. The adsorption energy, *E*_ads_, was calculated as follows:

$$E_{{{\text{ads}}}} = E\;({\text{adsorbed}}\;{\text{furan}}) - E\;({\text{furan}}) - E\;({\text{clean}}),$$

where *E* (adsorbed furan), *E* (furan), and *E* (clean) are the energies determined for furan adsorbed on a four-dimer cluster, a furan molecule, and a four-dimer cluster, respectively.

## Results and discussion

**Figure 1:**
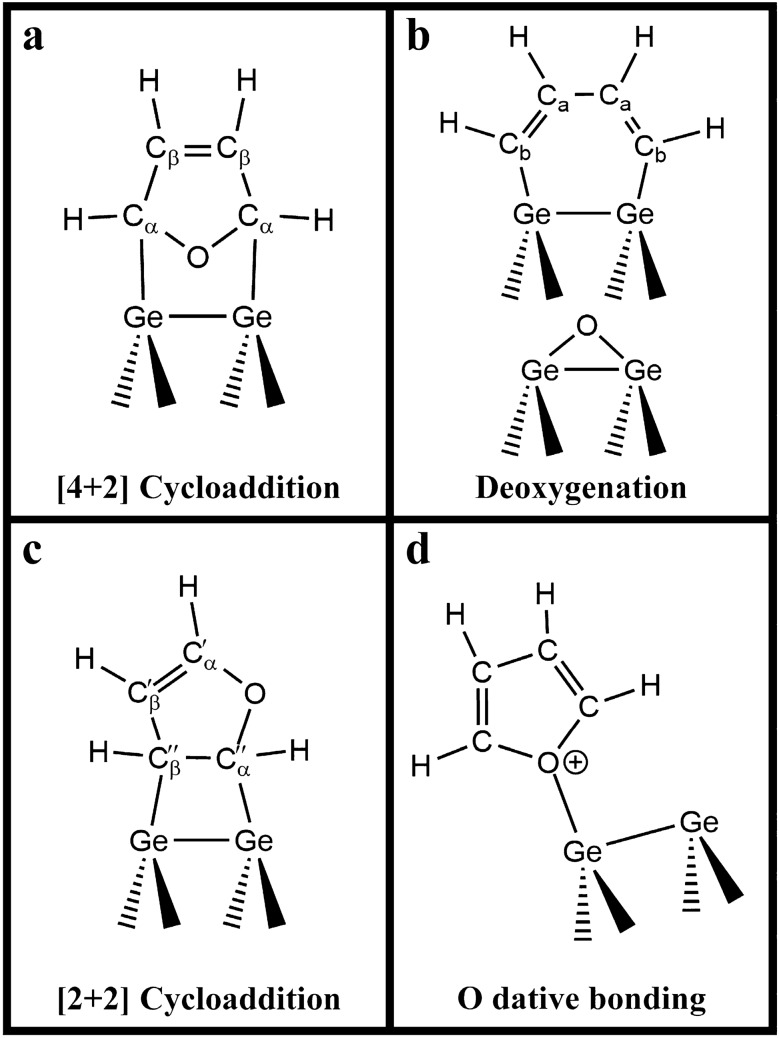
Four possible adsorption structures of furan on the Ge(100) surface: (**a**) [4 + 2] cycloaddition, (**b**) deoxygenation, (**c**) [2 + 2] cycloaddition, and (**d**) O dative bonded structures.

Furan has the reaction sites in its aromatic ring, namely an oxygen atom and the two C=C bonds. Thus, we anticipated several adsorption configurations for furan on the Ge(100) surface. Considering the previously reported reaction behaviors of the five-membered aromatic heterocyclic molecules of furan, thiophene, and selenophene on the Ge(100) and Si(100) surfaces^15–18,34–36^, four possible adsorption structures of furan on the Ge(100) surface were considered. Figure 1a,c show the adsorption features formed by the [4 + 2] and [2 + 2] cycloaddition reactions, respectively. In addition to the two cycloaddition products, Fig. 1b shows the deoxygenation adduct, in which an oxygen atom is transferred to a neighboring germanium dimer site with the formation of a Ge–(CH)_4_–Ge species. The molecularly adsorbed structure via O dative bonding is also described in Fig. 1d.

**Figure 2:**
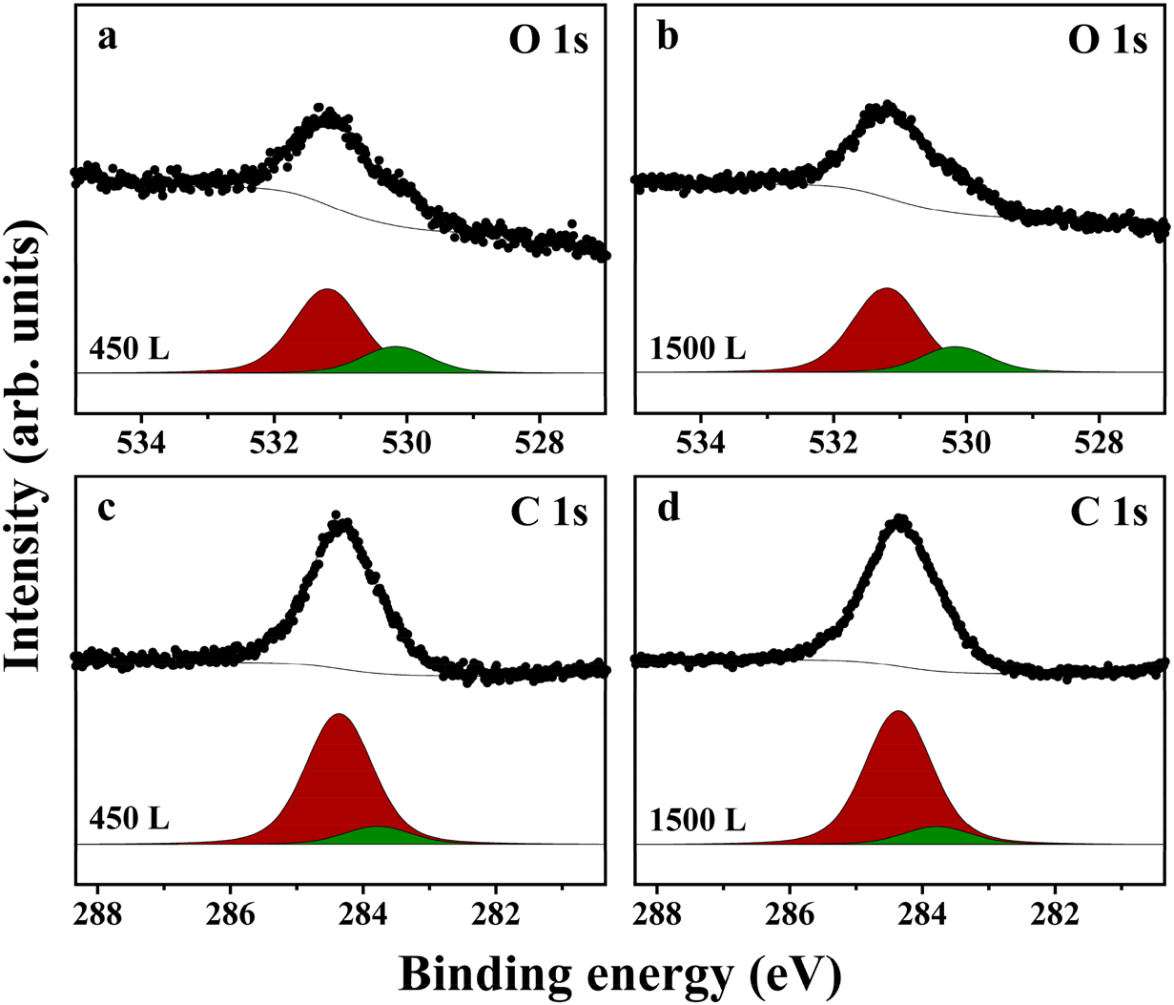
(**a**,**b**) O 1*s* and (**c**,**d**) C 1*s* core-level spectra obtained after the exposure of the Ge(100) surface to (**a**,**c**) 450 and (**b**,**d**) 1500 L of furan at room temperature. Solid black dots represent experimental data, while black lines represent the outcomes of the peak deconvolution process.

Figure 2 exhibits the O 1*s* and C 1*s* spectra acquired after the deposition of 450 and 1500 L (Langmuir) furan on the Ge(100) surface at room temperature. We found that the O 1*s* and C 1*s* spectra measured after the exposure of the Ge(100) surface to 450 L furan were similar to those measured after its exposure to 1500 L furan, indicating that the type of adsorption structures and their ratios did not change significantly at these coverages. In the O 1*s* spectra, two distinct peaks were observed at 530.2 and 531.2 eV owing to the two types of oxygen atoms present in the different chemical environments. Previous studies on the adsorption of CH_3_OH, C_4_H_8_O, and H_2_O on the Ge(100) surface have reported that peaks corresponding to a positively charged oxygen atom in the molecularly adsorbed species formed via O dative bonding appear at 533.1, 533.5, and 533.8 eV, respectively^12,37,38^. Hence, considering the binding energies of the peaks at 530.2 and 531.2 eV in the O 1*s* core-level spectra, we could exclude the formation of the O dative bonded configuration (Fig. 1d) by the adsorption of furan on the Ge(100) surface. The binding energies for the oxygen atoms in the O dative bonded structure of tetrahydrofuran and [4 + 2] cycloaddition structure of furan adsorbed on the Si(100) surface are 535.0 and 532.3 eV, respectively, with the difference being 2.7 eV^15,39^. By this logic, because the O 1*s* peak for the oxygen atom in the O dative bonded structure of tetrahydrofuran adsorbed on the Ge(100) surface occurs at 533.5 eV^12^, the O 1*s* signal for the oxygen atom in the [4 + 2] cycloaddition adduct of furan adsorbed on the Ge(100) surface may appear at approximately 530.8 eV, which is 2.7 eV lower than 533.5 eV. Therefore, we assigned the peak at 531.2 eV to an oxygen atom in the [4 + 2] cycloaddition configuration (Fig. 1a). In a previous study, the binding energy for an oxygen atom bound to two Ge atoms in the Ge–O–Ge configuration was reported to be 530.0 eV^23^. Through this result, we proposed that the peak at 530.2 eV was ascribable to the oxygen atom in the Ge–O–Ge form produced by the deoxygenation reaction (Fig. 1b). Next, based on the two adsorption structures ascertained from the O 1s spectra, to analyze the binding energies of the peaks present in the C 1*s* spectra, the carbon atoms were distinguished using a label (Fig. 1). Figure 2c,d show that two distinct peaks were observed at 283.8 and 284.4 eV in the C 1*s* spectra after peak deconvolution. With respect to the carbon atoms of the C=C bond present in the [4 + 2] cycloaddition and desulfurization structures formed by the adsorption of thiophene on the Si(100) surface, the binding energy for the carbon atoms unbound to the surface Ge atom has been reported to be 284.5 eV^18^. Therefore, the peak at 284.4 eV in the C 1*s* spectra was assigned to C_β_ and C_a_ because they are unbound to the surface Ge atom in the C=C bond (Fig. 1a,b). The peaks related to the carbon atom directly bonded to the surface Ge atom appear at 284.0–284.1 eV in the C 1*s* spectra^40–42^. Based on these results, we assigned the C 1*s* peak at 283.8 eV to C_b_. In a previous study on the adsorption of cyclopentene on the Ge(100) surface, the peaks associated with the carbon atoms bound and unbound to the surface Ge atom in the [2 + 2] cycloaddition product were observed at 283.5 and 284.2 eV, respectively^43^, with the difference being 0.7 eV. This result indicates that the binding energy of the C 1*s* peak is lowered by 0.7 eV if the carbon atom is bonded to the surface Ge atom. Because the binding energy of a carbon atom directly attached to a neutral oxygen in the Ge–(CH_2_)_4_–O–Ge species produced by the adsorption of tetrahydrofuran on the Ge(100) surface is 285.0 eV^12^, we ascribed the peak at 284.4 eV to C_α_ which is linked to both the oxygen and surface Ge atoms. Furthermore, we could reconfirm that our assignment of the peak at 283.8 eV to C_b_, which is bound to the surface Ge atom, was correct because its binding energy was 0.6 eV lower than that of the peak of C_a_ (284.4 eV). If a [2 + 2] cycloaddition structure (Fig. 1c) is formed by the adsorption of furan onto the Ge(100) surface, a peak must appear at a binding energy higher than 284.4 eV in the C 1*s* spectrum because the electron within C_α_′ is attracted by the oxygen directly bound to it owing to the difference in the Pauling electronegativities (PEs) of carbon (PE = 2.54) and oxygen (PE = 3.61)^44^. As shown in Fig. 2c,d, because no peak is observed at energies greater than 284.4 eV, we could rule out the formation of a [2 + 2] cycloaddition structure by the reaction of furan with the Ge(100) surface.

To confirm the validity of the two adsorption structures related to [4 + 2] cycloaddition and deoxygenation, as suggested by the analysis of the binding energies of the peaks observed in the O 1*s* and C 1*s* spectra, we examined the relative areas of the peaks in the spectra. Based on the peak areas of the two distinct peaks present in the O 1*s* spectra, we could infer the relative ratios of the two adsorption structures. Next, we calculated the expected relative proportion of each carbon atom present in the corresponding adsorption species. By comparing the relative ratios of the observed and expected C 1*s* peaks, we could confirm whether the assignments to the two proposed adsorption structures were reasonable. The ratios of the areas of the peaks detected at 531.2 and 530.2 eV in the O 1*s* spectra corresponded to the relative populations of the [4 + 2] cycloaddition and deoxygenation species, respectively. Their relative abundances were 76.1:23.9 at 450 L and 76.7:23.3 at 1500 L (Fig. 2a,b and Table 1). In addition to the ratios of the O 1*s* peaks, the relative percentages of the two C 1*s* peaks at 283.8 and 284.4 eV were determined to be 88.0:12.0 at 450 L and 88.4:11.6 at 1500 L, as shown in Fig. 2c,d and Table 1. The expected relative ratios of the C 1*s* peaks at 283.8 and 284.4 eV based on the number of carbon atoms present in the two adsorption structures determined by the O 1*s* peaks were calculated to be 88.0:12.0 at 450 L and 88.4:11.6 at 1500 L, which values exactly match the experimentally determined relative proportions of the two C 1*s* peaks (Table 1). Thus, the HRPES data confirmed the formation of two distinct adsorption species related to the [4 + 2] cycloaddition (major) and deoxygenation (minor) owing to the reaction of furan with the Ge(100) surface. Moreover, we expect that the formation of the deoxygenation structure following the adsorption of furan on the Ge(100) surface but not on the Si(100) surface is due to the different chemical reactivities of the two surfaces^45^.

**Table 1:** Relative proportions of the [4 + 2] cycloaddition and deoxygenation structures for furan adsorbed on the Ge(100) surface as determined from O 1*s* spectra (first two columns). Relative ratios of the two peaks observed in C 1*s* spectra (middle two columns). Last two columns show the expected relative ratios of the two C 1*s* peaks as calculated based on their relative ratios while assuming the [4 + 2] cycloaddition and deoxygenation structures determined based on the O 1*s* spectra.

Amount of furan exposed	Relative population ratios (%) determined from O 1*s* spectra (Fig. 1a,b)		Relative peak ratios (%) determined from C 1*s* spectra (Fig. 1c,d)		Expected relative C 1*s* peak ratios (%) inferred from the relative population ratios determined from the O 1*s* spectra	
	[4 + 2] Cycloaddition structure	Deoxygenation structure	Peak at 283.8 eV (C_α_/C_β_/C_a_)	Peak at 284.4 eV (C_b_)	Peak at 283.8 eV (C_α_/C_β_/C_a_)	Peak at 284.4 eV (C_b_)
450 L	76.1	23.9	88.0	12.0	88.0	12.0
1500 L	76.7	23.3	88.4	11.6	88.4	11.6

**Figure 3:**
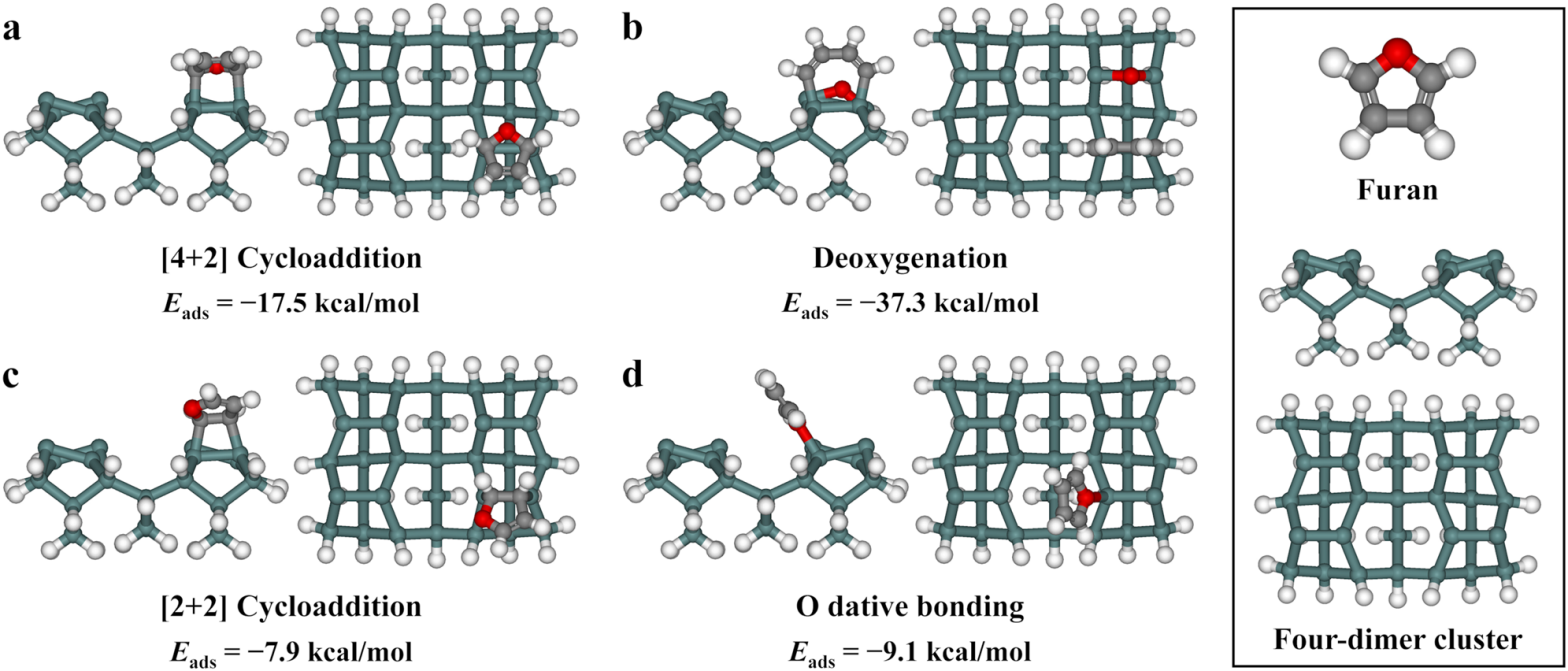
Calculated adsorption energies of the geometrically optimized structures for (**a**) [4 + 2] cycloaddition, (**b**) deoxygenation, (**c**) [2 + 2] cycloaddition, and (**d**) O dative bonding of furan on a four-dimer (Ge_35_H_32_) cluster. The right panel shows a furan molecule and the four-dimer cluster used in the DFT calculations. White, gray, red, and teal balls represent hydrogen, carbon, oxygen, and germanium atoms, respectively.

To determine whether the two adsorption structures identified by HRPES were energetically reasonable, DFT calculations were performed to obtain the adsorption energy (*E*_ads_) values for the four possible structures in Fig. 1. As shown in Fig. 3, the calculated *E*_ads_ values of the [4 + 2] cycloaddition and deoxygenation structures on a Ge_35_H_32_ cluster were − 17.5 and − 37.3 kcal/mol, respectively, which were more stable than the other two species with *E*_ads_ values of − 7.9 and − 9.1 kcal/mol. According to a previously reported DFT analysis performed for the adsorption structures of thiophene, which has a molecular structure similar to that of furan except for the heteroatom present in the ring, on a germanium cluster model, the [4 + 2] cycloaddition structure is thermodynamically favorable among the three possible adsorption structures, namely, those related to [4 + 2] cycloaddition, [2 + 2] cycloaddition, and S dative bonding^36^, which is consistent with the DFT data (Fig. 3). The authors suggested that [4 + 2] cycloaddition reaction is concerted and barrier-less because they did not identify a saddle point for it in the DFT calculations^36^. In addition, based on the calculated potential energy surface on the pathway from the [4 + 2] cycloaddition structure to the desulfurization structure of thiophene on a Ge_15_H_16_ cluster model, they concluded that the desulfurization adduct can be further produced from [4 + 2] cycloaddition through the transfer of a sulfur atom to an adjacent Ge dimer, with the *E*_ads_ values of the [4 + 2] cycloaddition and desulfurization species being − 18.3 and − 42.3 kcal/mol, respectively^36^. These values are similar to those of the comparable structures obtained by the DFT calculations in this study. Furthermore, the reaction from [4 + 2] cycloaddition to desulfurization has been reported previously in a DFT study on the adsorption of thiophene on the Si(100) system^34^. Therefore, we suggested that the [4 + 2] cycloaddition and deoxygenation structures are both produced by the adsorption of furan on the Ge(100) surface through a mechanism similar to that for thiophene.

## Conclusions

We investigated the adsorption structures of furan on the Ge(100) surface using HRPES and DFT calculations. By analyzing the binding energies and relative area ratios of all the peaks observed in the HRPES spectra, we confirmed the two distinct adsorption species formed by [4 + 2] cycloaddition and deoxygenation reactions. The DFT results were converged with the HRPES data, and they indicated that the formation of the [4 + 2] cycloaddition and deoxygenation structures via the adsorption of furan on the Ge(100) surface was reasonable. These findings regarding the reaction behavior of furan on the Ge(100) surface will further our understanding of the surface reactions of five-membered heterocyclic molecules.

## Data Availability

All data generated or analyzed during this study are included in this published article.
